# Hexane extract from *Lindera communis* roots: wound healing properties and membrane-disruptive activities against methicillin-resistant *Staphylococcus aureus*

**DOI:** 10.3389/fphar.2025.1528398

**Published:** 2025-02-11

**Authors:** Miaomiao Wang, Xian Hu, Liya Liu, Yi Zhong, Wanlin Li, Qing Zhang, Congli Xu, Chunlin Long

**Affiliations:** ^1^ Key Laboratory of Ecology and Environment in Minority Areas (Minzu University of China), National Ethnic Affairs Commission, Beijing, China; ^2^ College of Life and Environmental Sciences, Minzu University of China, Beijing, China; ^3^ Key Laboratory of Ethnomedicine (Minzu University of China), Ministry of Education, Beijing, China; ^4^ College of Ethnology and Sociology, Minzu University of China, Beijing, China; ^5^ Mass Spectrometry Imaging and Metabolomics (Minzu University of China), National Ethnic Affairs Commission, Beijing, China; ^6^ Baoshan Administration of Gaoligongshan National Nature Reserve, Baoshan, Yunnan, China; ^7^ Institute of National Security Studies, Minzu University of China, Beijing, China

**Keywords:** *Lindera communis*, antibacterial activity, methicillin-resistant *Staphylococcus aureus*, hexane extract, wound healing

## Abstract

**Introduction:**

The extensively used *Lindera communis* Hemsl. (Lauraceae) in traditional Chinese medicine has been specifically employed for wound healing and treating skin diseases in cattle and horses, suggesting its potential antibacterial properties. To explore the antibacterial activities of *L. communis* plants, we investigated the chemicals, antibacterial activities and wound healing and of the n-hexane fraction of *L. communis* roots (LCH).

**Methods:**

Our study included detecting phytochemical constituents, determining minimum inhibitory concentration (MIC) for different extract fractions, analyzing growth curves, assessing membrane integrity, monitoring potential changes in the membrane using scanning electron microscopy, and evaluating wound healing in rat excisional wounds.

**Results:**

Based on our findings, humulene-type sesquiterpenes, guaiane-type sesquiterpenes, and lauric acid were identified from the LCH, responsible for antibacterial and wound healing activities. The results are that LCH affected the growth of methicillin-resistant *Staphylococcus aureus* (MIC: 0.1 mg/mL) through morphological alterations and disrupting cell surface structures, causing membrane hyperpolarization and altering membrane integrity. This result was subsequently validated through SEM analysis and cytotoxicity against HaCaT cells (IC_50_ 1.83 ± 0.21 mg/mL). LCH also has exhibited remarkable effectiveness in healing rat excisional wounds, reinforcing its traditional use as a wound-healing agent.

**Discussion:**

The findings substantiate the scientific essence of traditional applications, while also exhibiting significant potential as a promising candidate for the development of innovative and readily accessible wound healing agents.

## Graphical Abstract

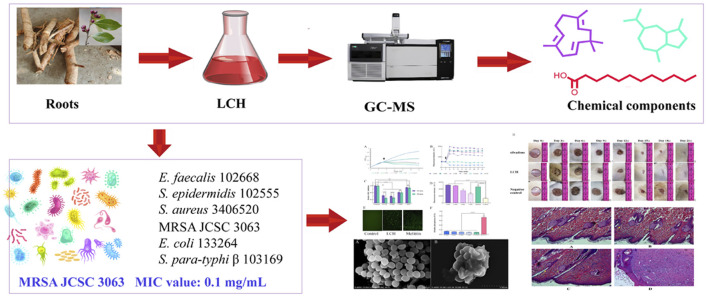

## 1 Introduction

The genus *Lindera* Thunb., encompassing approximately ninety-nine species within the Lauraceae family, is renowned for its aromatic leaves. It is primarily distributed across the warm regions of Asia and North America, where it dominates the dominant evergreen broad-leaved forests (Plants of the World Online: https://powo.science.kew.org/, accessed on March 20, 2024). *Lindera* species have multifaceted economic applications, including timber production, edible fruits, spices, and essential oils for perfumes and soaps (Editorial Committee of Flora Republicae Popularis Sinicae, 1982). These species also have a long history in traditional medicine for various ailments, including rheumatism, headaches, indigestion, and gastroenteritis. Numerous species are rich in natural compounds, particularly the roots of *L. aggregata* (Sims) Kosterm, which is a renowned TCM referred to as “wuyao” (Cao et al., 2015). Most *Lindera* plants produce complex secondary constituents, such as flavonoids, terpenoids, and steroids, and exhibits notable antibacterial activities (Cao et al., 2015).

*L. communis* Hemsl., the target species of this study is an evergreen plant native to central and southern China, Vietnam, Myanmar, India and southern Japan (Liao, 1996). The usage of this substance in Chinese traditional medicine dates back to ancient times due to its well-documented hemostatic, analgesic, and antipyretic properties. Additionally, its fruit can be used to extract aromatic oil for fragrance. The roots, branches and leaves have been traditionally employed as a remedy for wound healing and skin diseases in cattle and horses (Hu, 1996). This traditional knowledge suggests that the *L. communis* may possess antibacterial properties, which aligns with findings in the existing literature (Yang et al., 1999). Based on this, we hypothesize that *L. communis* possesses potential therapeutic properties for bacterial infections. The hexane extract from *Lindera* roots, known for its high activity within the *Lindera* genus, is being investigated for antimicrobial activity due to the different constituents such as sesquiterpenes.

The emergence of drug-resistant (MDR) microorganism has presented a serious threat to healthcare in recent decades and is responsible for various bacterial infections (Kupferschmidt, 2016). These strains, including MRSA, vancomycin-resistant *Enterococci*, carbapenem-resistant bacteria, and colistin-resistant Enterobacteriaceae, presents a significant menace to the wellbeing of the general public (Chambers and Deleo, 2009; Tacconelli and Cataldo, 2008; Walsh et al., 2011). As a result, the discovery of new antibacterial agents has become a priority. The utilization of natural constituents, particularly plant extracts and their components, has been extensively employed in bacterial infections (Sohn et al., 2004; More et al., 2017).

Therefore, we selected MRSA to investigate the antibacterial activity of LCH. Our study included the detection of phytochemical constituents, determination of the MIC of various fractions of *L. communis* extracts, investigation of the growth pattern of bacteria, examination of the integrity of cell membranes, observation of alterations in membrane potential, and assessment of the effectiveness against MRSA using scanning electron microscopy (SEM). Moreover, wound healing models were employed to evaluate the possibility and scientifically substantiate its uneventful application as a wound healing agent.

## 2 Materials and methods

### 2.1 Sample collection and preparation

The roots of *L. communis* were collected from Tengchong in Yunnan Province, China, in June 2022. A voucher specimen was expertly identified by Dr. Chunlin Long and has been archived in the Herbarium at MUC.

Briefly, the air-dried and powdered roots of *L. communis* (1.2 kg) were extracted with 95% EtOH (30 L × 3) at room temperature. The solvent was evaporated under reduced pressure, resulting in a brown residue (LCR, 276.2 g), which was then suspended in ddH_2_O (3 L) and subjected to sequential extractions with n-hexane (3 L × 3) and EtOAc (3 L × 3), ultimately yielding hexane-soluble fractions: the *n*-hexane-soluble (LCH) fraction (101.2 g), EtOAc-soluble (LCE) fraction (87.1 g) and H_2_O -soluble (LCW) fraction (67.3 g).

### 2.2 Chemical analysis

Sample (100 ± 2 mg) was put into the 20 mL headspace bottle, and 10 μL of 2-Octanol (10 mg/L stock in dH_2_O) was added as internal standard. All samples were analyzed by gas chromatograph system coupled with a spectrometer (GC-MS) and GC-MS analysis of each biological replicate was replicated three times. In SPME cycle of PAL rail system, the incubate temperature was controlled at 60°C. Preheat time is 15 min; Incubate time is 30 min; Desorption time is 4 min. GC-MS analysis was performed using an Agilent 7,890 gas chromatograph system coupled with a 5977B mass spectrometer. The system utilized a DB-Wax. Injected in Splitless Mode. Helium was used as the carrier gas, the front inlet purge flow was 3 mL min^−1^, and the gas flow rate through the column was 1 mL min^−1^. The initial temperature was kept at 40°C for 4 min, then raised to 245°C at a rate of 5°C min^−1^, kept for 5 min. The injection, transfer line, ion source, and quad temperatures were 250, 250, 230, and 150°C, respectively. The energy was −70 eV in electron impact mode. The mass spectrometry data were acquired in scan mode with the m/z range of 20–400, a solvent delay of 2.37 min. Chroma TOF 4.3X software of LECO Corporation and Nist database were used for raw peaks exacting, the data baselines filtering and calibration of the baseline, peak alignment, deconvolution analysis, peak identification, integration, and spectrum match of the peak area.

### 2.3 MIC and time-killing assay

The LCH was diluted in MHB medium at 37°C and incubated in a 96-well microtiter plate format for the MIC of LCH against *E. faecalis* (*Enterococcus faecalis* 102668), *S. epidermidis* (S*taphylococcus epidermidis* 102555), *S. aureus* (*Staphylococcus aureus* 3406520), *E. coli* (*Escherichia coli* 133264), *S. para-typhi β* (*Salmonella para-typhi β* 103169), and, Methicillin-resistant *Staphylococcus aureus* MRSA JCSC 3063 were cultured under aerobic condition. Through previous experimental validation, it was confirmed that dimethyl sulfoxide (DMSO), used as the solvent, exhibited no significant toxic effects on methicillin-resistant *Staphylococcus aureus* (MRSA) at a concentration of 2.5%. Different concentrations of LCH groups were solubilized using 2.5% DMSO, while the control group received an equivalent volume of 2.5% DMSO without LCH. MRSA was cultured in MHB at 37°C until at an OD_600_ of 0.3 and then treated with LCH at 0.1, 0.2, 0.4, and 0.8 mg/mL (1, 2, 4, and 8 MIC) (Epoch full-wavelength microplate reader, BioTek Company, United States). To ensure the consistency of experimental conditions, in addition to maintaining uniform drug administration protocols, we rigorously controlled other factors that could influence bacterial growth, such as temperature, humidity, shaker speed, and incubation time.

### 2.4 Membrane potential measured with DiOC2(3)

MRSA (logarithmic growth phase) was subjected to three washes with 5 mM HEPES. Following this, the bacterial were adjusted to achieve an OD_600_ of 0.5, thoroughly mixed, and subsequently incubated with DIOC2(3) (30 μM) (Thermo Fisher Scientific 66, Massachusetts, United States) for 20 min at 37°C. Following an incubation period, the bacterial cells were exposed to various concentrations of LCH (0.1, 0.2, 0.4, and 0.8 mg/mL) as well as CCCP at a concentration of 100 µM. Measurements were conducted in fluorimeter equipped with 530 nm excitation and 630 nm emission filters (Multifunctional enzyme marker, Espire, PerkinElmer, United States).

### 2.5 Resazurin assay

MRSA was cultured at 37°C until reaching an OD_600_ of 0.3 and subjected to treatment with LCH at 0.1, 0.2, 0.4, and 0.8 mg/mL, or 100 µM CCCP. Samples were collected at 10, and 30 min, diluted to an OD _600_ of 0.15, and then incubate with resazurin at 30°C. Fluorescence intensities were measured at 540 and 630 nm (Multifunctional enzyme marker, Enspire, PerkinElmer, United States).

### 2.6 Membrane integrity measured with PI

The MRSA (OD_600_ = 0.4) was treated with the LCH for 4 h, then incubated with 10 μg/mL PI until the fluorescence reading stabilized. The fluorescence intensity was measured at 535 nm (excitation) and 617 nm (emission) of (Multifunctional enzyme marker, Enspire, PerkinElmer, United States).

### 2.7 SYTOX green assay

The MRSA suspension (logarithmic growth phase, OD_600_ = 0.4) was washed three times with 0.85% NaCl, then treated with 4 MIC of LCH and melittin for 3 hours, and incubated with 3 µM of SYTOX green. Finally, the suspension was fixed on a 6-well plate and then observed using a fluorescence microscope (IX81 electric inverted microscope, Olympus, Japan) with an excitation wavelength of 485 nm.

### 2.8 Morphological observation of MRSA

Scanning electron microscopy (SEM) was used to observe the morphological changes in MRSA. MRSA were treated with 0.2 mg/mL LCH for 4 h, collected by centrifuge at 5,000 g for 10 min, and then fixed with 2%–5% glutaraldehyde overnight at 4°C. A gradient dehydration protocol was employed, sequentially immersing the samples in ethanol solutions with concentrations of 30%, 50%, 70%, 85%, 90%, 95% and finally, absolute ethanol (100%). The sample underwent drying, followed by deposition of a 10 nm-thick coating using an ion sputtering device. Subsequently, it was stored in a desiccator until examination under a scanning electron microscope operating at 20–0 kV (Hitachi S-4800, Tokyo, Japan).

### 2.9 AKP activity

The MRSA was washed three times with centrifuge, and the cell was adjusted to 10^6^ CFU/mL. Then, the bacterial was supplemented with LCH at 0.1, 0.2, 0.4, and 0.8 mg/mL and subjected to incubation at 37°C for a duration of 10 h. Following bacterial collected by centrifuge at 8,000 g for 10 min, and alkaline phosphatase was tested by AKP assay kit (Beyotime, Shanghai, China).

### 2.10 Mammalian cytotoxicity

The cell viability of LCH on HaCaT were assessed using the Cell Counting Kit-8 (CCK-8). Briefly, HaCaT cells were seeded in 96-well plates with 100 μL (2 × 10^5^ cells/well). Following incubation with varying concentrations of the tested substance, then tested with CCK-8 assay kit. Finally, we measured the OD value at 450 nm using a Microplate Reader (Beyotime, Shanghai, China).

### 2.11 Wound healing on the epithelialization

A total of 32 SD rats were procured from the Department of Experimental Animal Science, Peking University Health Science Center (SCXK(JING)2021-0013) and acclimated for 1 week prior to the experiment. The animals were housed in 22° ± 2° with natural light/dark cycles and provided clean water and chow. The rats with excision wounds were divided into four groups, each consisting of eight rats: the model M group (wounds + no treatment), the control (C) group (no wounds), the positive group (wounds + treated with 1% SSD (Silver sulfadiazine)), and the LCH group (excision wounds + treated with 10 mg LCH (red oil)). After 24 h of wound creation, LCH (red oil) 10 mg, 1% silver sulfadiazine (SSD) 0.1 g were topically applied to gently cover the wounded area once daily until complete wound healing. Wound area and wound contraction were monitored every 48 h until complete wound healing was achieved for all groups. All the wounds received daily standard wound cleansing with 0.9% normal saline prior to the topical application of treatments (0.1 g/unit wounded area [4 cm^2^]/day). The experimental procedure was carried out in compliance with the ethics requirements (ECMUC2021012AO).

### 2.12 Histology of healed tissue

Upon full wound healing, rats were euthanized approved by the ethical review of MUC. The sections of tissue were prepared using an established method (George et al., 1995). In brief, the healed tissues were initially fixed in 10% formalin, followed by progressive dehydration by graded ethanol, clearing in xylene, and subsequent embedding in paraffin wax for histological assessment. The tissue samples were sectioned into four-mm slices using a microtome, followed by staining with Harris hematoxylin and eosin (HE). The stained sections were then mounted onto microscopic slides, covered with coverslips, and subsequently examined using a light microscope. Images were acquired using Ultra View ERS confocal laser scanning microscopy (Nikon/OLYMPUS, E100/CX23, Japan).

### 2.13 Statistical analysis

All the data were subjected to ANOVA for multiple groups using GraphPad Prism 10.0 software (Prism GraphPad) (*P* < 0.05).

## 3 Results

### 3.1 GC-MS analysis

The phytochemical components present with their name, molecular formula, compound types, retention time (RT), as well as their relative abundance (Aera), which was quantified as the percentage of peak area detected by GC-MS. The results revealed that 49 compounds, based on comparisons with the NIST library. These compounds belong to different classes, including fatty acids, terpenes and other compounds. The chemical composition of the LCH is shown in Table 1, accounting for 85.68% of the total content. The volatile oils in the root hexane Extract of *L. communis* included characteristic detected components such as humulene-type sesquiterpenes, elemane-type sesquiterpenes, guaiane-type sesquiterpenes and Decanoic acid, ethyl ester (lauric acid), which is consistent with the phytoconstituents reported the review of *Lindera* species (Cao et al., 2015).

**TABLE 1:** Analysis of volatile components of LCH.

No.	Compounds	Molecular formula	Class	RT (minutes)	Aera
1	Humulene	C_15_H_24_	H-S	23.1	10.29%
2	Aciphyllene	C_15_H_24_	G-S	24.1	9.79%
3	Decanoic acid, ethyl ester	C_12_H_24_O_2_	fatty acid	22.4	6.22%
4	(1*R*,1a*R*,2a*S*,6*R*,6a*S*,7a*S*)-1,6,6a-Trimethyldecahydro-1,2a-methanocyclopropa[b]naphthalene	C_15_H_24_	sesquiterpene	22.6	5.84%
5	(-)-α Panasinsen	C_15_H_24_	sesquiterpene	25.2	4.33%
6	2,4-Di-tert-butylphenol	C_14_H_22_O	phenols	35.9	3.36%
7	Hexanoic acid, ethyl ester	C_8_H_16_O_2_	fatty acid	11.6	2.18%
8	Guaiol	C_15_H_26_O	G-S	31.9	2.14%
9	Nonanoic acid, ethyl ester	C_11_H_22_O_2_	fatty acid	19.9	2.11%
10	(2*R*,8*R*,8a*S*)-8,8a-Dimethyl-2-(prop-1-en-2-yl)-1,2,3,7,8,8a-hexahydronaphthalene	C_15_H_22_	E-S	26.4	2.11%
11	Calamenene	C_15_H_22_	sesquiterpene-Calamenene	26.7	2.06%
12	5,7-dimethyl-undecane	C_13_H_28_	fatty acid	13.5	1.85%
13	6,7-Dimethyl-1,2,3,5,8,8a-hexahydronaphthalene	C_12_H_18_	E-S	17.6	1.63%
14	Dihydro-3-methylene-5-methyl-2-furanone	C_6_H_8_O_2_	ester	26.6	1.60%
15	Calamenene	C_15_H_24_O	E-S	25.6	1.50%
16	4a,8-Dimethyl-2-(prop-1-en-2-yl)-1,2,3,4,4a,5,6,7-octahydronaphthalene	C_15_H_24_	E-S	23.2	1.40%
17	Propanoic acid	C_3_H_6_O_2_	fatty acid	20.1	1.33%
18	4a,5-Dimethyl-3-(prop-1-en-2-yl)-1,2,3,4,4a,5,6,7-octahydronaphthalen-1-ol	C_15_H_24_O	E-S	28.24	1.31%
19	1,4-Dimethyl-7-(prop-1-en-2-yl)decahydroazulen-4-ol	C_15_H_24_	G-S	34.1	1.30%
20	2-pentyl-Furan	C_9_H_14_O		11.5	1.18%
21	2-((2*R*,4a*R*,8a*S*)-4a-Methyl-8-methylenedecahydronaphthalen-2-yl) acrylaldehyde	C_15_H_22_O	E-S	37.5	1.10%
22	Pentanoic acid	C_5_H_10_O_2_	fatty acid	25.1	1.06%
23	(-)-Nootkatene	C_15_H_22_	E-S	25.6	1.04%
24	Heptanoic acid, ethyl ester	C_9_H_18_O_2_	fatty acid	14.5	1.01%
25	Epicubenol	C_15_H_26_O	E-S	32.1	0.99%
26	Myrtenol	C_10_H_16_O	monoterpene	25.9	0.98%
27	6,7-Dimethyl-1,2,3,5,8,8a-hexahydronaphthalene	C_12_H_18_		20.5	0.98%
28	(*E*)- Calacorene	C_15_H_20_	E-S	28.4	0.95%
29	delta-Amorphene	C_15_H_24_	E-S	25.1	0.90%
30	delta-Guaiene	C_15_H_24_	G-S	24.9	0.90%
31	2-methylene-5-(1-methylvinyl)-8-methyl-Bicyclo[5.3.0]decane	C_15_H_24_	G-S	21.6	0.90%
32	1-Naphthalenol	C_15_H_24_O	E-S	26.8	0.84%
33	1-Hexanol	C_6_H_14_O	fatty acid	15.9	0.76%
34	Hexadecane	C_16_H_34_	fatty acid	12.0	0.75%
35	Decane	C_10_H_22_	fatty acid	5.0	0.71%
36	Glaucyl alcohol	C_15_H_24_O	G-S	37.7	0.67%
38	(1*R*,3*E*,7*E*,11*R*)-1,5,5,8-Tetramethyl-12-oxabicyclo[9.1.0]dodeca-3,7-diene	C_15_H_24_O	H-S	30.9	0.64%
39	Dodecane	C_12_H_26_	alkane	10.5	0.62%
40	2-Ethylhexan-1-ol	C_8_H_18_O	fatty acid	18.8	0.61%
41	Terpineol	C_10_H_18_O	alcohol	23.8	0.60%
42	(*E*)-2-Undecene, 3-methyl	C_12_H_24_O_2_	alcohol	11.9	0.59%
43	Diethyl butanedioate	C_8_H_14_O_4_	ester	23.3	0.58%
44	Benzaldehyde	C_7_H_6_O	aldehyde	19.4	0.58%
45	(1*R*,4*S*,5*R*)-Verbenyl, ethyl ether	C_12_H_20_O	fatty acid	15.6	0.56%
46	3-methyl-Butanoic acid	C_5_H_10_O_2_	fatty acid	23.4	0.55%
47	5-Hexen-2-one	C_6_H_10_O	ester	8.6	0.54%
48	Pinocarveol	C_10_H_16_O	monoterpene	22.8	0.54%
49	10-epi-gamma-Eudesmol	C_15_H_26_O	phenolic acids	34.0	0.54%

Note: RT: retention time, E-S, elemane-type sesquiterpene: G-S, guaiane-type sesquiterpene: H-S, humulene-type sesquiterpene.

### 3.2 MIC and time-killing assay

The roots of *L. communis* were extracted using 95% ethanol, and the resulting crude ethanol extracts was fractionated to obtain n-hexane, EtOAc, and water fractions. As shown in Table 2, all of the extracts presented bactericidal activity against bacteria. The antimicrobial activity of LCH against MRSA JCSC 3063 (MIC: 0.1 mg/mL). The fractions exhibited limited efficacy against Gram-negative bacteria.

**TABLE 2:** The screening of antibacterial activity.

Bacteria	MIC (mg/mL)					
	*E. faecalis* 102668	*S. epidermidis* 102555	*S. aureus* 3406520	MRSA JCSC 3063	*E. coli* 133264	*S. para-typhi β* 103169
G^+^/G^−^	G^+^	G^+^	G^+^	G^+^	G^−^	G^−^
LCR	2.875	0.65	0.65	0.65	>100	>100
LCW	3.56	0.79	1.56	1.56	>100	>100
LCE	6.25	6.25	12.5	12.5	>100	>100
LCH	0.2	0.1	0.1	0.1	>100	>100
Vancomycin	ND	ND	0.62 × 10^−3^	0.62 × 10^−3^	ND	ND
Kanamycin	ND	ND	ND	ND	12.5	12.5

ND: not detected.

**FIGURE 1:**
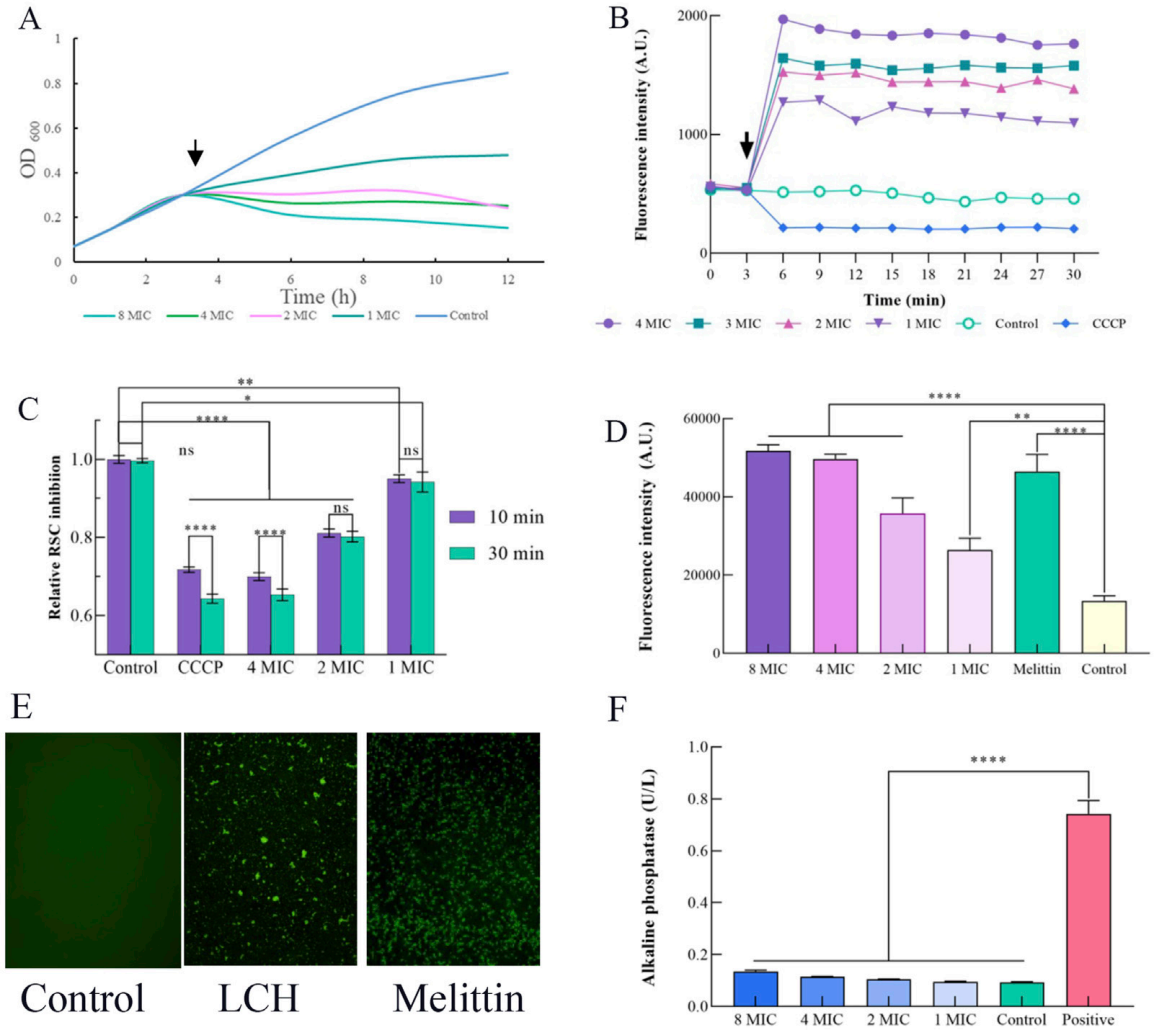
Effects of LCH on the cell envelope of MRSA (0.1, 0.2, 0.4, and 0.8 mg/mL that is 1× MIC, 2× MIC, 4× MIC and 8× MIC). **(A)** Growth curve. Impact of LCH on MRSA growth. Arrow indicates the time point of LCH addition. **(B)** Measurement of membrane potential using the fluorescent dye DiOC(3)5 with 0.1, 0.2, 0.4, and 0.8 mg/mL LCH. The arrow denotes the time point of LCH addition, and CCCP was used as a positive control. **(C)** Resazurin assay. Assessment of respiratory chain activity through resazurin reduction to resorufin with the treatment of 0.1, 0.2, 0.4, and 0.8 mg/mL LCH. CCCP served as a positive control. **(D)** Staining with PI. The fluorescent dye serves as an indicator for the presence of substantial membrane pores or significant membrane disruption in MRSA with the treatment of 0.1, 0.2, 0.4, and 0.8 mg/mL LCH, while melittin was employed as a positive control. **(E)** The fluorescence emission of MRSA with the LCH was detected through SYTOX Green binding to nucleic acid. The use of SYTOX Green showed that LCH at 1 mg/mL for 4 h significantly inhibited membrane rupture in MRSA, while no fluorescence increase was seen in the control group. Melittin was used as a positive control. **(F)** Influence of LCH on the cell wall. AKP activity of MRSA treated by LCH. *P < 0.05, **P < 0.01***, *P* < 0.001****, *P* < 0.0001. Data are expressed as mean ± SD, n = 3. LCH: Hexane Extract from *Lindera communis* Roots.

As shown in Figure 1A, the impact of LCH on the growth of MRSA is detected. Arrow marks are the time point of LCH addition. The bactericidal activity of LCH was equivocal after 1 h exposure at concentrations of 1× MIC and 2× MIC, while a pronounced bactericidal effect was observed with a marked reduction in CFU following 1 h exposures to doses of different multiples of MIC values.

### 3.3 Membrane potential measured with DiOC2(3)

DIOC2(3) is a potentiometric probe known for its affinity to visualized the change of membrane permeability. Disruption of membrane permeability results in the release of the probe into the surrounding medium, thereby inducing a substantial enhancement in fluorescence. DIOC2(3) is a widely acknowledged membrane potential probe that specifically localizes within the bacterial membrane to detect alterations in membrane potential (Minderman et al., 1996; Aqawi et al., 2021). We investigated whether LCH disrupts the membrane potential using the probe DiOC2(3) (Figure 1B). Indeed, our results revealed hyperpolarization of cells, in contrast to the depolarization induced by the proton ionophore CCCP (Figure 1B), showing that LCH may give an ion channel, a membrane pore, or even potentially influence the respiratory chain. And further studies will be conducted to confirm this hypothesis (Saeloh et al., 2018).

### 3.4 Resazurin assay

To ascertain the impact of LCH on respiratory chain, we evaluated the decrease of resazurin, a widely used indicator for quantifying cellular reductive capacity (Abu-Amero and Bosley, 2005; Müller et al., 2016). Certainly, after incubation with LCH, there was only a slight reduction in the reductive capacity of MRSA, suggesting a relatively modest impact on the electron transport chain (Figure 1C). The observed effect exhibited a comparable magnitude to that elicited by CCCP, known for its role in decoupling the electron transport chain, and was closer to the control group (Figure 1C). Consequently, the influence of LCH on the maintenance and generation of the proton motive force can be deduced.

### 3.5 Membrane integrity measured with PI

PI was employed to further investigate and probe the membrane-disrupting effects of LCH, which was utilized to assess the integrity of the cell membrane through binding to exposed nucleic acids in a fluorescent fashion (Lin et al., 2017). Melittin, used as a positive control, resulted in substantial increases in fluorescence intensity, signifying the disruption of cell membranes. This effect was further validated through the use of the fluorescent indicator PI is capable of cellular entry through pores or in cases of severe membrane breaches (Wenzel et al., 2013). After treatment with LCH, a notable PI influx was observed at concentrations of 1×, 2×, 4× and 8× MIC, closely resembling the results obtained with Melittin (Figure 1D). This indicated that LCH may either disrupt bacterial cell membranes or create small breaches in them. Thus, the PI assay corroborates the findings from the membrane potential study, where significant fluorescence intensity was observed across different concentrations. It is speculated that LCH exerts its antibacterial effects by affecting the membrane.

### 3.6 SYTOX green assay

Most satisfyingly, the SYTOX Green assay yielded consistent results with the fluorescence intensity, which was observed in the PI assay (Figure 1E). When LCH was treated with 1 mg/mL LCH for 4 h, the fluorescence was clearly observed in the SYTOX Green assay. The control group, in contrast, exhibited no change in fluorescence, whereas the melittin group induced a sharp increase in fluorescence intensity under identical conditions. This result aligns with the findings of the PI assay, further confirming that LCH disrupts the cell membrane (Lebaron et al., 1998).

### 3.7 AKP activity

Alkaline phosphatase (AKP), located within the space between the cellular membrane and wall, is released into the extracellular milieu upon compromise or damage to the cell wall (Diao et al., 2018). Therefore, the AKP activity serves as an indicative measure of the cell wall’s integrity. As depicted in Figure 1F, AKP activity in the C group and the LCH-treated groups at 1×, 2×, 4×, and 8× MIC remained relatively stable even after a 10 h incubation period, whereas the standard group exhibited a significant 5.39-fold increase in AKP activity, which indicates that the permeability of the MRSA cell wall remained largely unchanged following treatment with LCH.

### 3.8 Morphological observation of MRSA

**FIGURE 2:**
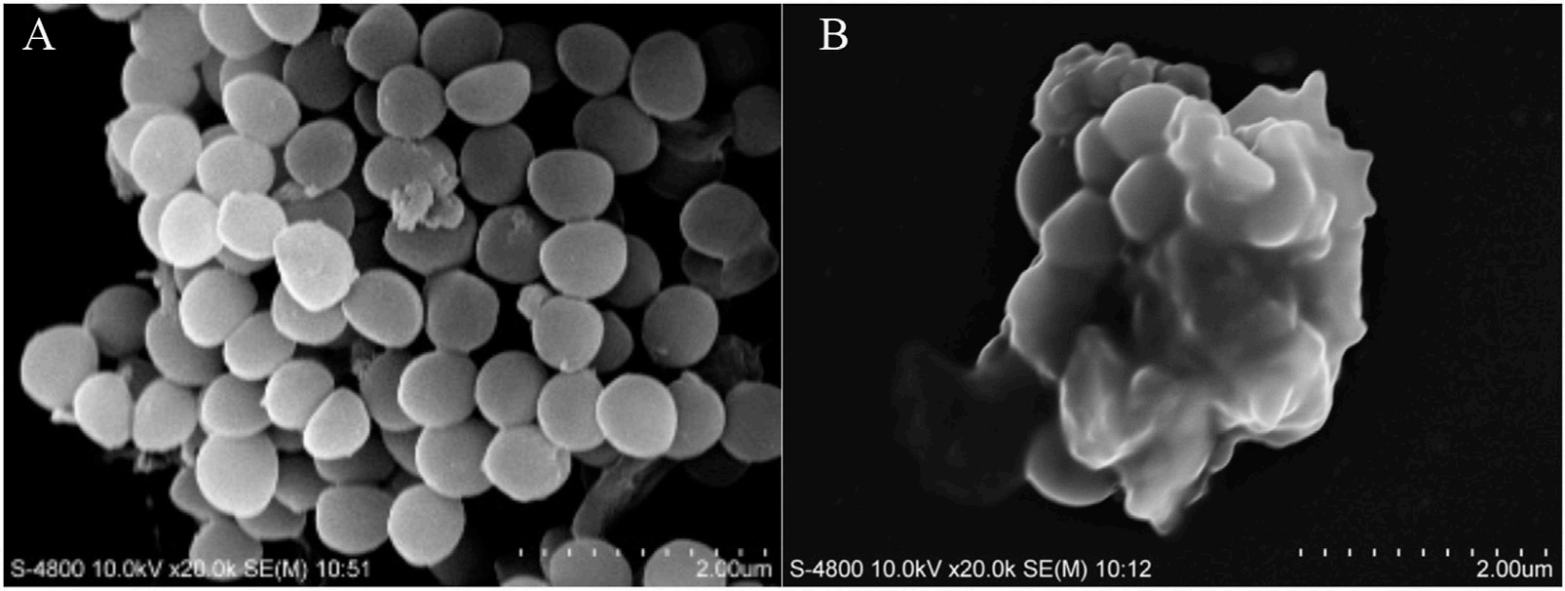
Morphological observation of MRSA by SEM. **(A)** Control. **(B)** 2× MIC (0.2 mg/mL).

The morphological alterations in MRSA induced by LCH were observed using SEM. As illustrated in Figure 2, the cell surface of the control group appeared smooth and round, with well-defined edges, intact structure, and uniform short rods. After a 4 h incubation with 0.2 mg/mL of LCH, significant morphological alterations were observed, characterized by distinct ruptured membranes and the collapse of cell surface structures. The observed alterations in cell morphology strongly indicate that the potent biological activities of LCH primarily result from its ability to disrupt membrane integrity.

### 3.9 Mammalian cytotoxicity

An assessment of the toxicities of LCH was conducted on the HaCaT cell lines (Human immortal keratinocyte lines). The IC_50_ value against HaCaT cells was 1.83 ± 0.21 mg/mL, Which is far higher than the MIC agaisnst MRSA and safety with an appropriate dosage.

### 3.10 Wound healing on the epithelialization

**FIGURE 3:**
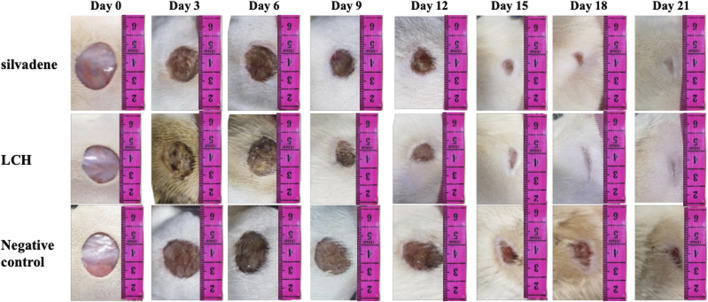
The effect of LCH on wound contraction was monitored every 72 h during treatment.

After establishing wounds model in rats, receiving daily treatments subsequently, it was observed that the untreated excisional-wounded rats had a longer period of epithelialization compared to 1% SSD group. Additionally, LCH treatment shortened the period of epithelialization compared to the M group (Figure 3). The wound contraction rate was observed to be slower in the Mrats compared to the 1% SSD group. However, treatment with LCH group significantly improved the wound contraction rate compared to the model group (Figure 3). Meanwhile, the control group exhibited noticeable pus formation from day 6–12, while the LCH-treated group did not. Previous studies have demonstrated the potential of *Lindera* extracts as analgesic agents due to their remarkable anti-inflammatory effects and pain-relieving properties in traditional medicine (Cao et al., 2015). It showed that antibacterial, noninflammatory and antinociceptive effects have resulted in the wounding healing.

### 3.11 Histology of healed tissue

**FIGURE 4:**
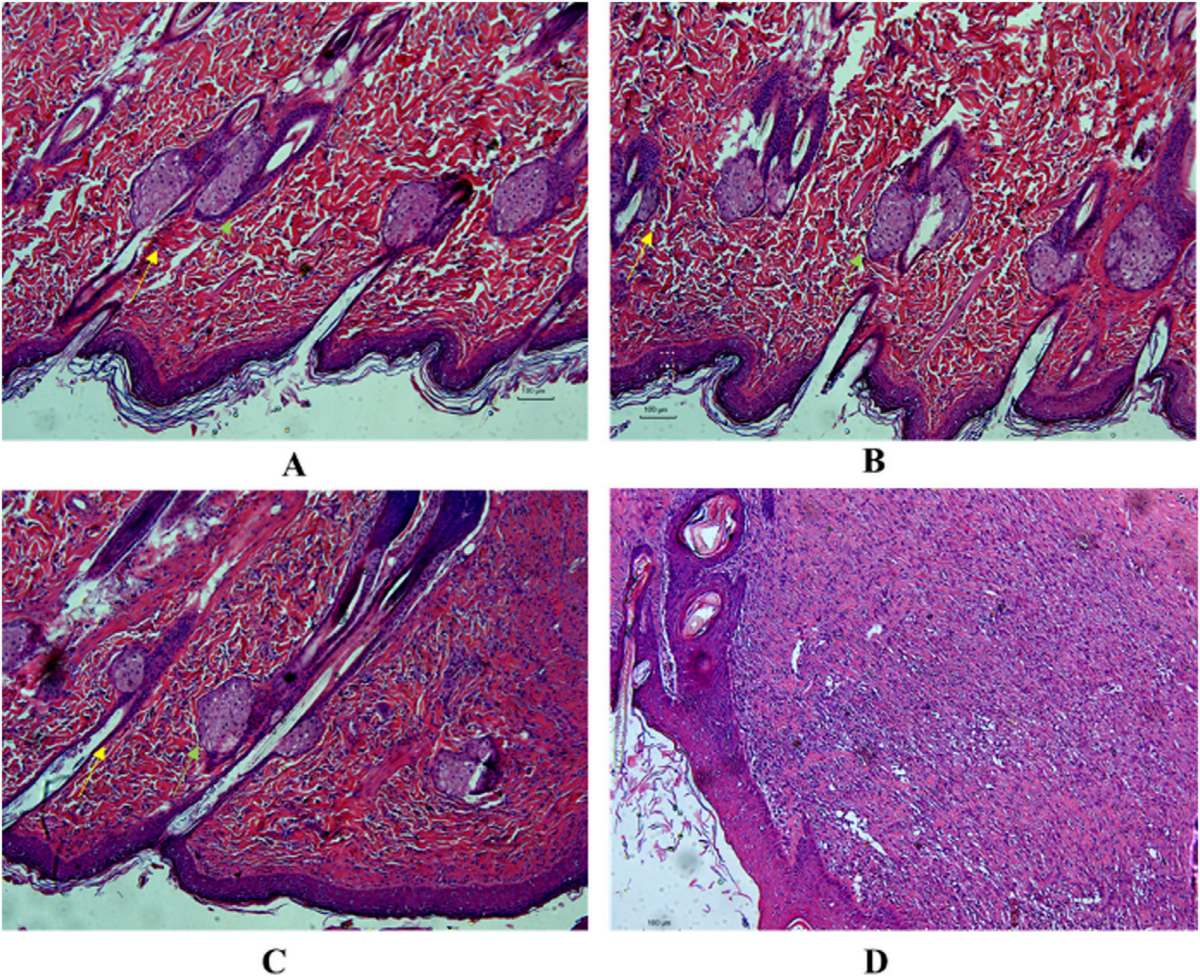
Histological section displays the regenerated epidermis and dermis in the healed wound tissue. **(A)** Control, **(B)** 1% SSD (0.1 g), **(C)** LCH (10 mg), **(D)** Model.

The control group showed variable-sized hair follicles, large sebaceous glands, and loose granulation tissue with limited inflammatory cell infiltration (Figure 4A). The positive control group showed a thin epidermis and loose granulation dermis with widespread eosinophilic collagen fibers, moderate inflammatory cell infiltration, coagulation within the hair follicle, fewer hair follicles, and sebaceous glands (Figure 4B). The model group exhibited a thickened epidermis and compact fibrotic granulation dermis with mild eosinophilia of collagen fibers, accompanied by significant infiltration of inflammatory cells and minimal presence of sebaceous glands (Figure 4D). The LCH group exhibited a thickened epidermis and a loosely arranged granulation dermis with moderate inflammation and sparse sebaceous glands (Figure 4C). The histomicrographs indicate that LCH demonstrates a significant decrease in the infiltration of inflammatory cells compared to the model, suggesting its potential as an effective anti-inflammatory and antibacterial agent.

## 4 Discussion

The mixture and the isolated compounds from *Lindera* hexane extract exhibit potential therapeutic activity against various diseases. The extract of *Lindera* has demonstrated significant antifungal and antibacterial properties and these antibacterial activities can be attributed to low polar compounds, such as sesquiterpenes, monoterpenes and fatty acids (Cao et al., 2015; Lv et al., 2023; Tao et al., 2024). Before the antibacterial experiment, we compared the antibacterial activities of various plant parts (roots, stems, leaves, and fruits) and found the roots to have the highest activity. Further tests on root fractions revealed the n-hexane fraction as the most active. Literature review (Yan et al., 2009) indicated that antibacterial components in *Lindera* root extracts are likely present in essential oils. Thus, we used SPME GC-MS for analysis. This aligns with reported effective antibacterial components in Lauraceae plants or Lindera (Wang et al., 1999; Kim et al., 2009; Yan et al., 2009; Joshi et al., 2010). Based on prior studies and fraction activity screening, we speculate that the antibacterial components are low-polarity compounds.

The major compounds detected in root LCH of *L. communis* includes the following groups: humulene-type sesquiterpenes, elemane-type sesquiterpenes, guaiane-type sesquiterpenes and lauric acids and others. Characterizations of GC-MS revealed the presence of humulane-type sesquiterpenoids, widely distributed in plants and microbes, which possessed antibacterial for *B. fragilis* (MIC 2 μg/mL) (Al-Taweel et al., 2004; Djabou et al., 2013; Jiao et al., 2018; Jang et al., 2020). The guaiane-type sesquiterpenes were also detected by GC-MS and the active constituents derived from medicinal plants are believed to yield enhanced and targeted pharmaceuticals. Guaiane-type sesquiterpenoids were identified based on their molecular weight and are known for their antibacterial, anti-inflammatory, and anticancer effects (Guo et al., 2023). In addition, fatty acids and their monoglyceride derivatives, such as lauric acid, show strong antimicrobial activities (Borrelli et al., 2021). After detection, the humulene-type sesquiterpenes, guaiane-type sesquiterpenes and lauric acid may be responsible for the antibacterial activities. Then we conducted assessments of antibacterial activity against MRSA and other common bacteria. In our present study, LCH exhibited antibacterial activities solely against MRSA (MIC = 0.1 mg/mL). It is anticipated that our future research will elucidate the primary active ingredients in greater detail, rather than solely relying on literature reviews and SPME GC-MS analysis.

To enhance comprehension LCH’s antibacterial mechanism, we assessed its time-kill kinetics, which demonstrated that LCH efficiently achieved reductions with different dose drugs within 15 h, displaying a concentration-dependent and time-dependent antibacterial activity pattern. Notably, many antibiotics target the cell membrane, membrane potential, and cell wall (Sani and Separovic, 2016; Epand et al., 2016). It is suggested that LCH may primarily target cytoplasmic membranes, as membrane composition is likely a key factor in the varying sensitivities of different bacterial species to LCH.

With the assistance of the DiOC2(3) probe, significant alterations in fluorescence intensity were observed upon treatment of MRSA with LCH. The resazurin assay further confirmed that LCH disrupted the membrane potential through affecting the respiratory chain of MRSA cells. Fluorescence probe PI and SYTOX Green, which bind to nucleic acids, along with fluorescence imaging technology, were to investigate the impact of LCH on the enhancement of membrane permeability (Strahl and Hamoen, 2010). As a result, they consistently showed detectable fluorescence intensity after bacterial treatment with 0.1–0.2 mg/mL LCH. However, LCH did not significantly increase AKP activity, even at 8 x MIC of LCH. SEM analysis of LCH-treated MRSA revealed changes in cell morphology, including noticeable perforations in the cell membrane. These consistent findings indicate that LCH inhibits MRSA by disrupting the cell membrane. The findings of the time-killing assay could be explained, where the bactericidal activity of LCH became evident after 1 h of exposure at all concentrations, and maintained persistent bactericidal efficacy throughout the entire 15-h duration. Therefore, these informative findings collectively substantiate the conclusion that the bactericidal activity of LCH is attributed to aberrations occurring at the cellular membrane.

A key characteristic of an ideal antibiotic is its harmlessness to the host. To evaluate this, cytotoxicity assays were conducted using HaCaT cells, determining an to be 1.83 ± 0.21 mg/mL with a therapeutic index of 0.1 μg/mL. Additionally, we assessed the impact of the LCH wound healing formula on the epithelialization period, which demonstrated significant effects. Histological analysis of the healed tissue also confirmed these findings. These results validate the scientific basis of the traditional knowledge regarding LCH.

## 5 Conclusion

Based on our findings, humulene-type sesquiterpenes, guaiane-type sesquiterpenes and lauric acid were identified as the main chemical constituents of LCH. At the same time, the LCH possessed excellent antibacterial activity against G^+^ bacteria especially the MRSA (MIC value: 0.1 mg/mL). The time-killing curve and SEM were employed to assess the efficacy of LCH against MRSA and effectively suppress its growth. We further investigated the impact of LCH on the membrane of MRSA and hypothesized that it exerted a profound influence on MRSA growth by inducing significant morphological alterations, leading to the disruption of cell surface structures, hyperpolarization of the membrane, and perturbations in membrane integrity. That is, the mode of action of the substance involves membrane perturbation as one of its mechanisms. LCH exhibited antibacterial efficacy against G^+^ bacteria with low minimal cytotoxicity. Meanwhile, LCH has exhibited wound-healing efficacy and bacteriostatic effects in excisional wounds in rats, confirming the potential use of *L. communi*s root as a wound-healing agent. All the findings presented in this study collectively demonstrate that LCH exhibits significant inhibitory effects on the growth of MRSA by disrupting its membrane. These results suggest that LCH holds great promise as a candidate for the advancement of innovative and readily available wound healing agents, and it proves the scientific essence of traditional uses.

## Data Availability

The original contributions presented in the study are included in the article material, further inquiries can be directed to the corresponding authors.
